# Prevalence of hepatitis C virus infection among prisoners in Iran: a systematic review and meta-analysis

**DOI:** 10.1186/s12954-018-0231-0

**Published:** 2018-05-09

**Authors:** Masoud Behzadifar, Hasan Abolghasem Gorji, Aziz Rezapour, Nicola Luigi Bragazzi

**Affiliations:** 1 Department of Health Services Management, School of Health Management and Information Sciences, Iran University of Medical Sciences, Tehran, Iran; 2 Health Management and Economics Research Center, Iran University of Medical Sciences, Tehran, Iran; 3 Department of Health Sciences (DISSAL), School of Public Health, University of Genoa, Genoa, Italy

**Keywords:** Hepatitis C virus, Prevalence, Prisoners, Iran, Meta-analysis, Systematic review

## Abstract

**Background:**

Hepatitis C virus (HCV) is one of the major public health problems both in developed and developing countries. Prison represents a high-risk environment for prisoners, in that it is characterized by high-risk behaviors such as injecting drug use (IDU), tattooing, unprotected sexual intercourses, or sharing syringes. The aim of this study was to quantitatively evaluate the prevalence of HCV among Iranian prisoners conducting a systematic review and meta-analysis.

**Methods:**

We searched different scholarly databases including Embase, PubMed/MEDLINE, ISI/Web of Sciences, the Cochrane library, Scopus, CINAHL, and PsycINFO as well as Iranian bibliographic *thesauri* (namely, Barakatns, MagIran, and SID) up to December 2017. The Newcastle Ottawa Scale (NOS) was used to assess the quality of the studies included. HCV prevalence rate with its 95% confidence interval (CI) was estimated using the DerSimonian-Laird random-effects model, with Freeman-Tukey double arcsine transformation. Egger’s regression test was used to evaluate publication bias.

**Results:**

Finally, 17 articles were selected based on inclusion and exclusion criteria. Overall, 18,693 prisoners were tested. Based on the random-effects model, the prevalence of HCV among Iranian prisoners was 28% (CI 95% 21–36) with heterogeneity of *I*^2^ = 99.3% (*p* = 0.00). All studies used an ELISA test for the evaluation of HCV antibodies. The findings of this study showed that the highest prevalence rate (53%) was among prisoners who inject drugs.

**Conclusion:**

The findings of our study showed that the prevalence of HCV among Iranian prisoners is dramatically high. Managing this issue in Iran’s prisons requires careful attention to the availability of health facilities and instruments, such as screening, and harm reduction policies, such as giving sterile syringes and needles to prisoners. An integrated program of training for prisoners, prison personnel and medical staff is also needed to improve the level of health condition in prisons.

## Background

Hepatitis C virus (HCV) is one of the major public health problems both in developed and developing countries, leading to acute and chronic hepatitis [1]. It is estimated that 71 million people in the world are suffering from chronic HCV, and about 399,000 people die each year due to cirrhosis and liver cancer [2]. The prevalence of HCV has been computed to be approximately 0.6% in the general population [3] and 0.5% among blood donors in Iran [4].

Prison represents a high-risk environment for prisoners, in that it is characterized by risky behaviors such as injecting drug use (IDU), tattooing, unprotected sexual intercourses, or sharing syringes. For example, IDU represents a major risk factor, with 52% of 11.7 million people injecting drugs worldwide living with HCV [5]. Furthermore, inadequate access to preventive interventions and diagnostic measures increases the risk of transmitting infections. HCV, like other pathogens, such as hepatitis B virus (HBV) and human immunodeficiency virus (HIV), is transmitted through blood [6, 7]. HCV infection represents the main cause of liver cancer and can affect the outcome of liver transplantation [8, 9]. Liver disease is a major cause of death among inmates and has recently surpassed HIV. Compared to other subjects, prisoners are more likely to develop HCV and to die of its complications [10–12]_._

According to the latest data available from the “Global State of Harm Reduction” report and to a comprehensive review of the global disease burden in prisoners, it has been estimated that approximately 15.1% of 10.2 million people incarcerated at any given time are living with HCV [5, 13]. More in detail, several studies have been conducted to determine the prevalence of HCV worldwide, reporting different findings. For example, 22.7% of prisoners in Spain [14], 6.9% in Sri Lanka [15], 17.7% in Turkey [16], 2.4% in Brazil [17], and 10.1% in the USA [18] have been found to suffer from HCV infection.

Precise epidemiological figures related HCV prevalence are fundamental in the health sector for policy- and decision-makers in each country to inform health policies in an evidence-based way [19]. In recent years, research has been conducted to determine the prevalence of HCV in Iran in order to estimate HCV infection among prisoners. The knowledge of the epidemiology of HCV among Iranian prisoners can effectively guide the design and implementation of ad hoc harm reduction programs. The aim of this study was to quantitatively evaluate the prevalence of HCV among Iranian prisoners conducting a systematic review and meta-analysis.

## Methods

A protocol for the current systematic review and meta-analysis was devised in accordance with the “Preferred Reporting Items for Systematic Reviews and Meta-Analyses” (PRISMA) guidelines [20] and prospectively registered in the PROSPERO database (identification ID: CRD42018082336).

Two authors independently searched for articles related to HCV infection among Iranian prisoners, mining scholarly databases such as Embase, PubMed/MEDLINE, ISI/Web of Sciences, the Cochrane library, Scopus, CINAHL, and PsycINFO. Iranian bibliographic *thesauri* were searched as well (namely, Barakatns, MagIran, and SID), with no language restrictions or time filter (searches performed from inception until December 2017). The following strategy was used: (“Prevalence” OR” Epidemiology” OR “Frequency”) AND (“Hepatitis C virus” OR “HCV” OR “Hepatitis “OR “Viral hepatitis”) AND (“Prison” OR “Prisoner” OR “Inmates” OR “Jails” OR “Incarceration “OR “Pre-trial detention” OR “Juvenile detention facilities”) AND “Iran.” The reference list of each potentially eligible study was also reviewed and hand-searched for increasing the chance of getting relevant investigations.

### Inclusion criteria

The inclusion criteria were as follows: (i) studies investigating the prevalence of HCV among Iranian prisoners; (ii) studies providing clear data and the possibility of calculating the prevalence; (iii) studies designed as observational studies (that is to say, a variety of designs were considered eligible including cross-sectional, case-control, prospective, or retrospective investigations); (iv) studies written in Persian and English; (v) studies that used validated diagnostic tests such as enzyme-linked immunosorbent assay (ELISA), polymerase-chain reaction (PCR), and recombinant immunoblot assay (RIBA); and (vi) studies that reported serological HCV data.

### Exclusion criteria

Exclusion criteria were as follows (i) studies not conducted among Iranian prisoners; (ii) studies not reporting sufficient data to estimate the prevalence rate; (iii) studies designed as editorials, letters to editor, commentaries, expert opinions, case-series, case-reports, reviews, or conference abstracts; and (iv) studies reporting overlapping/updated data.

### Quality assessment

The Newcastle Ottawa Scale (NOS) was used to assess the quality of the studies included. Based on the scores, studies were classified into three categories, namely, studies characterized by high risk (score in the range 1–3), moderate risk (score in the range 4–6), and low risk (score in the range 7–9).

### Data extraction

Two authors independently extracted relevant information from the articles, including the surname of the first author, the location of the study, the year of publication, the sample size, the mean age of the participants, the diagnostic test used for HCV screening, the reported prevalence, the number of people living with HCV, and the duration of imprisonment.

### Statistical analysis

HCV prevalence with its 95% confidence interval (CI) was estimated using the DerSimonian-Laird random-effects model [21] with Freeman-Tukey double arcsine transformation [22]. To quantitatively assess heterogeneity among articles, *I*^2^ test was used [23]. In case of statistically significant heterogeneity, in order to investigate its possible sources, meta-regression analyses were performed based on the year of publication, sample size, and the duration of the imprisonment. Furthermore, sensitivity analyses were performed to ensure stability of the findings. Egger’s regression test was used to evaluate publication bias [24]. Based on sample size, quality of studies, and length of conviction, subgroup analyses were conducted. Figures with *p* value < 0.05 were considered statistically significant. Data were analyzed using STATA Ver.14 (Stata Corp, College Station, TX, USA).

## Results

**Fig. 1:**
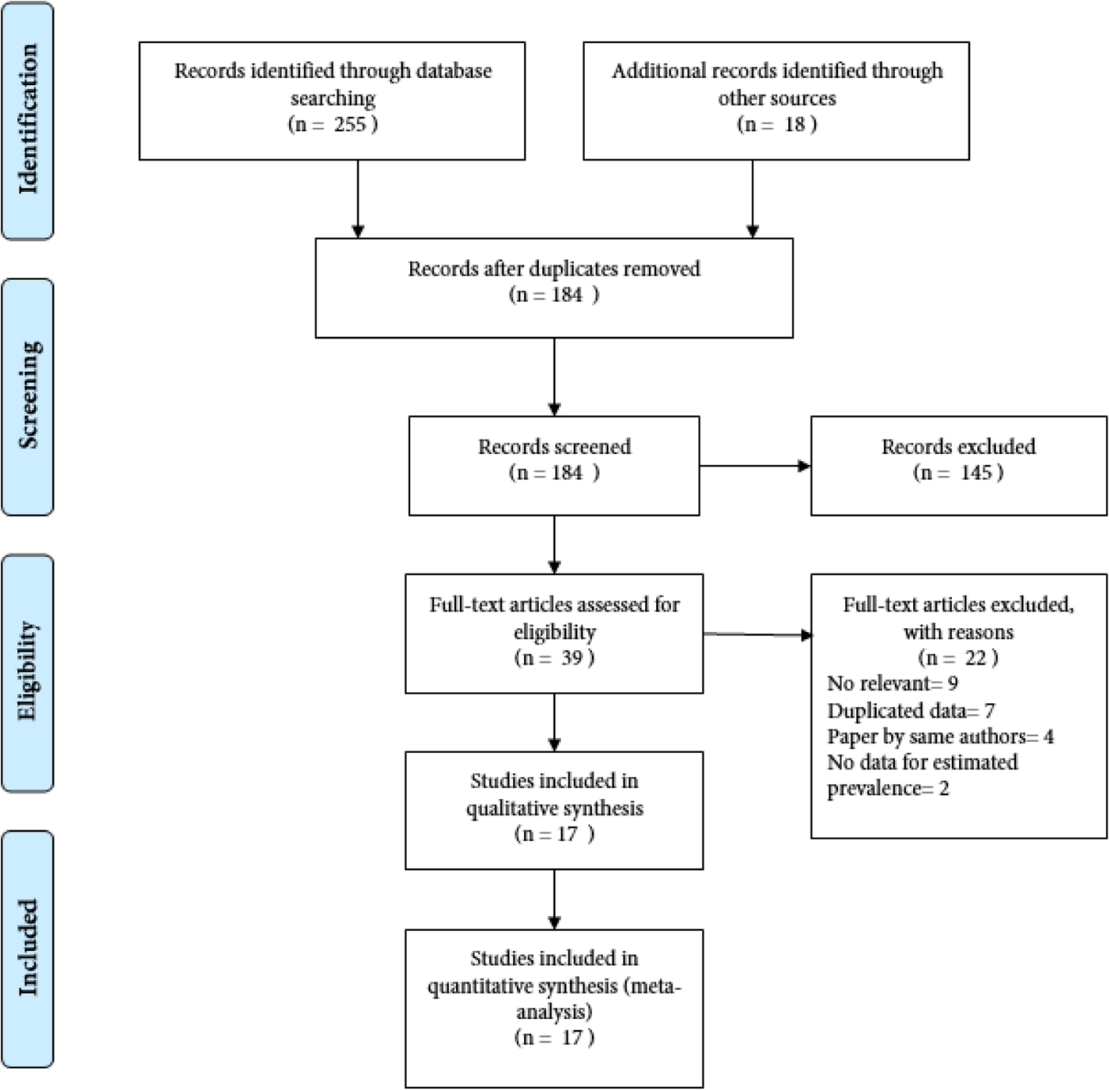
The process of search and selection of studies

We identified an initial list of 273 articles, which resulted in 184 items after deleting 89 duplicates. By reviewing the title and/or abstract of these 184 articles, 145 unrelated articles were discarded. The full texts of 39 articles were reviewed, and finally 17 articles were selected based on inclusion and exclusion criteria. Figure 1 shows the process of searching and selecting studies.

In the current systematic review and meta-analysis, 18,693 prisoners were tested. Studies were published between 2004 and 2016. The sample size was between 101 and 8630. All studies used an ELISA diagnostic test for the evaluation of HCV antibodies. Concerning the quality of the studies, one study was found to be at high risk, six at moderate risk, and ten at low risk. Table 1 shows the main characteristics of the studies included.

**Table 1:** The characteristics of studies included

First author	Publication year	Province	Age (mean ± SD)	Sample size	Prevalence (%)	Diagnostic test	Quality of studies
Rowhani-Rahbar	2004	Razavi Khorasan	32.8 ± 8.9	110	59.4	ELISA	High
Mohammad Alizadeh	2005	Hamedan	NA	427	30	ELISA	Medium
Zakizadeh	2006	Mazandaran	NA	312	30.80	ELISA	High
Mohtasham Amiri	2007	Guilan	34.7 ± 8.9	460	45.40	ELISA	Medium
Pourahmad	2007	3 provinces	NA	1431	34.70	ELISA	High
Azarkar	2007	South Khorasan	34.1 ± 11.7	400	7.80	ELISA	High
Asgari	2008	10 provinces	NA	8630	37.85	ELISA	High
Zakizad M	2009	Mazandaran	NA	312	30.80	ELISA	Medium
Tajbakhsh	2009	Chaharmahal and Bakhtiari	25.8	600	12.66	ELISA	Low
Davoodian	2009	Hormozgan	35.4 ± 8.4	249	64.80	ELISA	High
Azarkar	2010	South Khorasan	34.7 ± 12	358	8.10	ELISA	High
Kassaian	2012	Isfahan	32.6	943	41.60	ELISA	Medium
Naghili	2012	East Azerbaijan	31.3 ± 10	192	29	ELISA	High
Nokhodian	2012	Isfahan	34.54 ± 11.2	163	7.40	ELISA	High
Ziaee	2014	South Khorasan	34.7 ± 11.4	881	7.70	ELISA	Medium
Alasvand	2015	6 Providences	37 ± 13	2120	12.90	ELISA	Medium
Khajedaluee	2016	Razavi Khorasan	53.37 ± 54.9	1114	24.50	ELISA	High

**Fig. 2:**
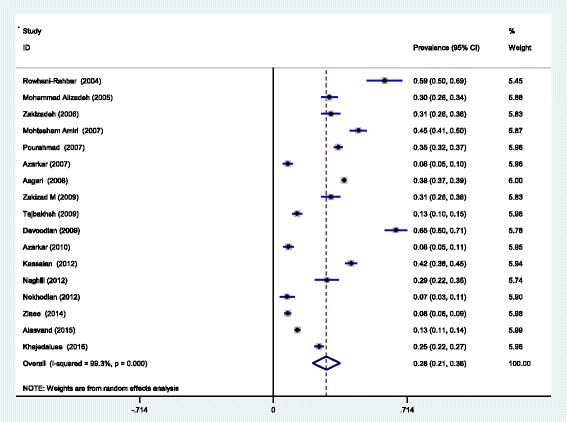
The overall prevalence of HCV in prisoners Iranian based on the random-effects model

Based on the random-effects model, the prevalence of HCV among Iranian prisoners was 28% (CI 95% 21–36) with heterogeneity of *I*^2^ = 99.3% (*p* = 0.00), as shown in Fig. 2.

**Fig. 3:**
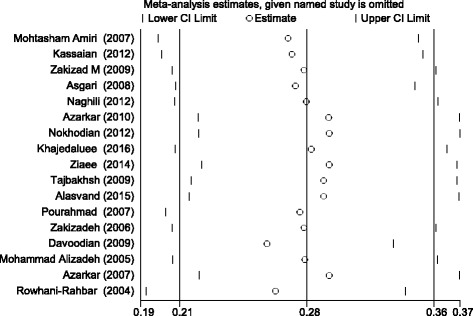
The sensitivity analysis of the prevalence of HCV in prisoners Iranian based on the random-effect model

To ensure the stability of the results, the sensitivity analysis was performed in order to examine the impact of each study on the overall prevalence. Before and after this analysis, the overall prevalence did not change and confirmed the stability of the findings (Fig. 3).

Subgroup analysis was performed based on variables such as quality, sample size, and length of conviction. Table 2 shows the results.

**Table 2:** The results of subgroup analysis

Items	Prevalence 95% CI	*I*^2^ (%)	*P*
Quality of studies			
High	13% (10–15)	–	–
Intermediate	28% (16–40)	99.1	0.00
Low	30% (20–40)	99.2	0.00
Sample size			
≤ 400	29% (16–43)	98.6	0.00
> 400	27% (18–37)	99.5	0.00
Prisoner type			
Prisoners who inject drugs	53% (42–64)	94.6	0.00
Prisoners who make use of drugs	25% (11–38)	97.5	0.00
General (non-risk)	19% (10–29)	99.5	0.00

To evaluate possible heterogeneity sources among studies, meta-regression analyses were carried out based on the year of publication, sample size, and duration of imprisonment. Table 3 shows the results. The prevalence was borderline significant based both on the duration of conviction (*p* = 0.073) and the year of study publication (*p* = 0.074).

**Table 3:** The results of meta-regression

Variables	Coef	SE	*P* value	Adjusted *R*^2^ (%)
Publication year	− 0.022	0.011	0.074	14.30
Sample size	6.38	0.000	0.78	− 6.31
Duration of incarceration (years)	0.108	0.049	0.073	36.01

**Fig. 4:**
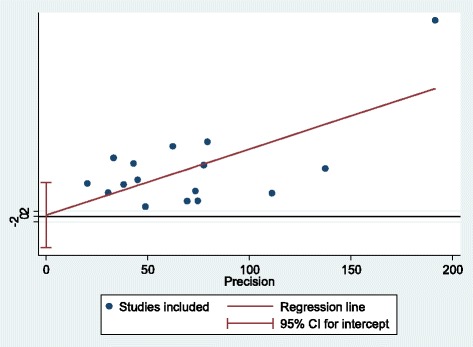
The publication bias based on the Egger’s test

Various risk factors for HCV among Iranian prisoners have been reported including history of traditional phlebotomy, blood transfusions, tattooing, razor sharing, surgery, history of dental operations, sharing a syringe/needle, drug use, unprotected sex, sexually transmitted infections, alcohol consumption, piercings, IDU, history of cupping, dialysis, and abortion. According to available data, we calculated the odds ratio (OR) for some of these factors (Table 4). Egger’s regression test showed that there was no publication bias for the selected studies (*p* = 0.94) (Fig. 4).

**Table 4:** Odds ratio (OR) for HCV-related risk factors

Items	Number of studies	Odds ratio (95% CI)	*I*^2^ (%)	*P*
Injection of drugs	8	3.54 (2.28–5.49)	88.6	0.00
Tattooing	6	2.59 (1.74–3.86)	81.7	0.00
Having shared a syringe/needle	4	4.06 (0.70–23.51)	95.4	0.00
History of transfusion	4	1.61 (0.91–2.86)	55	0.00
Use of drugs	3	1.16 (0.38–3.54)	94.3	0.00

## Discussion

To the best of our knowledge, this study was the first and most comprehensive systematic review and meta-analysis that examined the prevalence of HCV among Iranian prisoners. Comprehensive search, the use of a checklist to assess the risk of studies, meta-regressions, sensitivity analysis, and publication bias assessment are among the strengths of this study.

HCV infection among inmates is a very serious problem that healthcare professionals in each country have to face. Given that there are about 10 million prisoners around the world [25], attention to HCV prevention should be a public health priority in prisons in all developed and developing countries [26].

The findings of this study showed that the prevalence of HCV among Iranian prisoners was 28% (CI 95% 21–36). This rate is higher than the prevalence in the general population [3]—0.6% (CI 95% 0.4–0.8%)—and blood donors in Iran [4]—0.5% (CI 95% 0.4–0.6%).

Compared to international studies, this rate is higher than the prevalence reported among prisoners in Italy (22.4%) [27], in Brazil (2.4%) [17], and in France (4.8%) [28], and lower than the prevalence reported in California (34.3%) [29] and in Indonesia (34.1%) [30].

The causes of the difference in the prevalence among the studies carried out in different countries can be the type of prison population surveyed on the basis of significant risk factors such as IDU or the history of imprisonment and the conduct of high-risk sexual behaviors or other high-risk behaviors.

Prison health conditions vary in different parts of the world, and in many countries, there is still no definite policy to further improve the health of prisoners. On the other hand, it seems that screening conditions are not adequately provided for many of them at the time of imprisonment. The rules governing the legal system of the countries make it possible to create complex constraints for screening HCV. Sometimes, it is, indeed, difficult to reach a balance between ensuring adequate health standards and public safety, for example guaranteeing to prisoners continuity of healthcare and access to healthcare facilities (such as screening centers), before and during the transfer to custodial settings. On the other hand, there are also financial problems in many sectors, such as the health and legal systems of the countries, which have led to the unfeasibility of screening all inmates [5]. Ensuring timely diagnostic tests for HCV would be, indeed, too much expensive. Even though numerous studies have assessed the cost-effectiveness of screening prisoners for HCV, there is a dearth of information specifically concerning Iran. Furthermore, despite the fact that HCV is very common in prisoners and threatens their health status and conditions, there is still no screening program in Iran as well as in many countries [27]. Over the past years, policies have been developed in Iran to improve the health situation in Iran’s prisons, but these efforts have not been sufficient and there is still a huge gap to be filled [31].

The findings of this study showed that the highest prevalence (53%) was among prisoners who inject drugs. This finding is in line with other published investigations, including a Scottish study [32]. In a meta-analysis study that evaluates HCV in prisoners, the prevalence of IDU was estimated to be 64% [10]. The most important risk factor associated with HCV is, indeed, IDU, and this is a common danger in all prisons in the world. However, because of the lack of the implementation of proper harm reduction policies, it still remains a major problem for prisoners [33].

Studies show that IDU-related practices in Iran are on the rise [34]. Prisoners who inject drugs are more susceptible to infections such as HCV, HBV, and HIV than others [35]. Unfortunately, in recent decades, drug use has increased in Iran, and the pattern of drug use has changed, with an increasing trend in IDU [36]. In our findings, the OR for HCV for prisoners who inject drugs compared to other prisoners was 3.54. Findings from various studies indicate that prisoners are potentially susceptible to HCV [17, 37, 38]. The OR in prisoners who had a history of sharing syringes/needles was 4.06 in comparison with other people. The lack of access to needle and syringe programs in prisons has led to a very high incidence of HCV [39]. Officials should identify prisoners who inject drugs to control HCV infection at the very beginning of their arrival, and provide them with sterile needles and syringes to prevent the spread of infection to other prisoners. The harm reduction-based training given to prisoners can also have an effective role in reducing the infection. In a study conducted in European countries, sterile syringes and needles were provided to prisoners, and they also received adequate training. Results showed that the use of syringes by other prisoners was reduced and no new cases HCV were reported [40].

Tattooing in prisons is another major health concern, due to the use of non-sterile devices leading to transmission of infections such as HCV, HBV, and HIV. In a meta-analysis study published in 2017, tattoos are considered as one of the most important risk factors for HCV transmission [41]. In the current study, those with a history of tattooing had a 2.59 higher risk than those without a history of tattooing. Two cohort studies in Australia have reported a significant relationship between tattooing and HCV infection [42, 43]. Prison authorities should provide harm reduction interventions for prisoners to help reduce any harms associated with these activities. A study conducted in seven European countries refers to the role of harm reduction programs and the improvement of health of prison population and the reduction of HCV [44].

The results of meta-regression analyses showed that there is a borderline significant relationship between the prevalence rate of HCV among Iranian prisoners and the year of studies publication. Since 2005, health policy- and decision-makers in the country have decided to formally adopt a harm reduction policy. Within this policy, prisoners who make use of drugs such as opioids can be legally treated with methadone. A strategy for making available sterile syringes and needles to prisoners who inject drugs was also considered [36]. However, despite the implementation of this policy, HCV infection among Iranian prisoners remains a challenge [45].

According to the findings of meta-regressions, the prevalence of HCV among Iranian prisoners has increased with time and length of conviction. The findings of a study carried out in Brazil seem to confirm this finding [17]. Prisoners are familiar with high-risk behaviors in prison over time, and in many cases, they may experience them. The results of a study in Ireland show that at least 21% of prisoners have experienced the first injection in jails [46].

However, this study has some limitations that should be mentioned: the major drawback is given by the presence of statistically significant heterogeneity among studies [47]. Furthermore, for many provinces of Iran, there is a dearth of information as no studies have been conducted. Moreover, data available did not allow comparisons to be performed based on gender.

## Conclusions

In conclusion, health policy- and decision-makers in Iran need good evidence to improve public health status in prisons. The findings of our study showed that the prevalence of HCV among Iranian prisoners was 28%. This prevalence is dramatically high. Managing this issue in Iran’s prisons requires careful attention to the availability of health facilities and instruments, such as screening, and harm reduction policies, such as making needle and syringe programs available to prisoners. An integrated program of training for prisoners, prison personnel, and medical staff is also needed to improve the level of health condition in prisons.

## Abbreviations

CI: Confidence interval
ELISA: Enzyme-linked immunosorbent assay
HBV: Hepatitis B virus
HCV: Hepatitis C virus
HIV: Human immunodeficiency virus
IDU: Injecting drug use
NOS: The New Castle Ottawa Scale
OR: Odds ratio
PCR: Polymerase-chain reaction
PRISMA: Preferred Reporting Items for Systematic Reviews and Meta-Analyses
RIBA: Recombinant immunoblot assay
